# Proteome-Wide Mendelian Randomisation Study of Adverse Perinatal Outcomes

**DOI:** 10.1007/s10519-025-10233-1

**Published:** 2025-09-24

**Authors:** Emily R. Daubney, Christopher Flatley, Liang-Dar Hwang, David M. Evans

**Affiliations:** 1 Institute for Molecular Bioscience, The University of Queensland, Brisbane, Australia; 2 The Frazer Institute, The University of Queensland, Woolloongabba, QLD 4102 Australia; 3 MRC Integrative Epidemiology Unit, University of Bristol, Bristol, UK

**Keywords:** Mendelian randomization, Proteome-wide, Perinatal outcomes

## Abstract

Despite significant advances in pre- and postnatal care over the last century, adverse pregnancy related events still occur frequently. We used Mendelian randomisation (MR) to investigate potential causal effects of the mother’s and fetal blood proteome on pregnancy related outcomes including birthweight, placental weight, preeclampsia, and sporadic miscarriage. We generated a list of genetic instruments to act as proxies for plasma proteins by combining two recent large protein GWAS (4719 proteins *N* = 35,559 individuals; 4775 proteins *N* = 10,708 individuals). We identified 1724 proteins with valid *cis*-pQTLs for use as genetic instruments. We identified evidence for causal relationships (MR Bonferroni corrected *p*_*Bonferroni*_ < 2.90 × 10^−5^) involving fetal effects and/or maternal effects on birthweight and preeclampsia. Increased levels of PSG7 and BCMA and decreased levels of VLCAD, INHBB, and PLCG1 in the fetal circulation were potentially causal for increased birthweight. Similarly, increased levels of LIMA1 and decreased levels of VLCAD, FBLN3, and galectin-4 in the maternal circulation were potentially causal for increased birthweight. Decreased levels of SERPINE2 and SIGLEC6 were potentially causal for increased risk of preeclampsia. We did not find any significant effects of proxied maternal or fetal proteins on placental weight or sporadic miscarriage, perhaps due to the smaller size of their GWAS meta-analyses. Our results implicate several proteins that may be involved in the aetiology of perinatal phenotypes that will need to be replicated in independent datasets.

**Supplementary Information:**

The online version contains supplementary material available at 10.1007/s10519-025-10233-1.

## Introduction

Despite significant advances in pre- and postnatal care over the last century, adverse pregnancy related events still occur frequently. The global stillbirth rate is approximately 13.9 per 1,000 total births (Hug et al. 2021), whilst the estimated risk of miscarriage is approximately 15% of all recognised pregnancies (with actual miscarriage numbers likely to be higher) (Farrell and Owen 1996). Likewise, preeclampsia, a major pregnancy-associated hypertensive disorder which can result in maternal and neonatal mortality, has an incidence of 2 to 8% of all pregnancies (Macedo et al. 2020). Whilst maternal and fetal genetic factors and a range of environmental factors have been implicated in these and other related conditions, in many cases, the reasons behind a large fraction of adverse pregnancy related events are unclear (Honigberg et al. 2023; Juliusdottir et al. 2021; Laisk et al. 2020; Reynoso et al. 2024; Stanley et al. 2020; Warrington et al. 2019). Understanding the molecular pathways underlying these adverse events may provide opportunities to pharmacologically modify the pathways involved and correspondingly decrease maternal/fetal morbidity and mortality.

Genome wide association studies (GWAS) represent a hypothesis free way of identifying molecular mechanisms underlying common complex traits and diseases. To date GWAS have had considerable success in elucidating the genetic aetiology of adverse perinatal outcomes, with the largest GWAS of birthweight (Juliusdottir et al. 2021; Warrington et al. 2019), miscarriage (Laisk et al. 2020), and pre-eclampsia (Honigberg et al. 2023) identifying 190, four, and eighteen robustly associated loci respectively. However, the majority of the heritability has yet to be explained for these traits (Maher 2008). GWAS typically lack power to detect genuinely associated variants at genome-wide levels of significance (*p* < 5 × 10^−8^) due to the small effect sizes underlying most complex trait and disease loci, and the extensive multiple testing burden associated with testing millions of genetic markers across the genome. In addition, most associations identified by GWAS lie in non-coding parts of the genome, making it difficult to determine the functional genes responsible for the statistical associations.

Recent years have witnessed the development and widespread availability of scalable affinity-based proteomic techniques which are able to measure thousands of protein targets simultaneously. Large scale GWAS of serum protein levels assayed by these technologies have resulted in the identification of hundreds of *cis*- and *trans*- protein quantitative trait loci (pQTLs) underlying variation in protein levels (Ferkingstad et al. 2021; Pietzner et al. 2021; Sun et al. 2023). This has in turn led to the emergence of protein-wide association studies (PWAS) where (usually) *cis*-pQTLs are used as genetic instruments to proxy serum protein levels, and test for association with traits of interest. PWAS have several potential advantages over GWAS and traditional observational epidemiological studies where protein levels are simply correlated with disease status. First, relative to GWAS, the burden due to multiple testing is reduced. Second, several *cis*-QTLs may explain more of the variation in protein levels than single genetic variants and hence power to detect association may be increased. Third, *cis*-pQTLs provide a functional mechanism linking genetic and trait variation, a goal of GWAS. Finally, PWAS studies harness the principles of Mendelian randomisation (MR) to inform on a potential causal relationship between circulating proteins and the disease/trait of interest. Specifically, since PWAS utilise genetic variants which are subject to Mendel’s Laws of Segregation and Independent Assortment, the results produced by these studies should be less prone to confounding and reverse causality than the results from traditional observational epidemiological studies using proteomic data (Davey Smith and Ebrahim 2003).

The present study aimed to use protein-wide two sample MR to investigate potential causal relationships between circulating proteins and a range of adverse perinatal outcomes and related traits relevant to pregnancy (Beaumont et al. 2023; Ferkingstad et al. 2021; Honigberg et al. 2023; Laisk et al. 2020; Pietzner et al. 2021; Warrington et al. 2019). Genetic instruments were generated by combining the results of two large-scale proteomic GWAS with potential causal relationships investigated for birthweight, placental weight, preeclampsia, and sporadic miscarriage.

## Materials and Methods

### Data Sources

#### pQTL GWAS

This project investigated circulating protein levels, as the exposure of interest, for potential causal relationships with birthweight, placental weight and a number of adverse perinatal outcomes. To obtain valid MR instruments (*cis*-pQTLs) to proxy circulating protein levels, two large cohort proteome GWAS were combined (Ferkingstad et al. 2021; Pietzner et al. 2021). Both proteome GWAS measured protein levels with the SomaScan multiplex aptamer assay (version 4).

Ferkingstad et al. (2021) released publicly available GWAS summary statistics for 4,907 aptamers measuring 4,719 proteins, in an Icelandic population sample (*n* = 35,559). Samples collected were the combined efforts of two different studies, the Icelandic Cancer Project (52% of participants) and deCODE genetics, Reykjavík, Iceland (48% of participants). The average participant age was 55 years (s.d = 17 years) with 57% being female. The GWAS results concern rank-based inverse normal transformed protein measures corrected for age, sex, and sample age. Correction for population stratification was performed by dividing test statistics by the estimated linkage disequilibrium (LD) score regression intercept.

Pietzner et al. (2021) released publicly available GWAS summary results statistics for 4,979 aptamers measuring 4,775 proteins, from a population of European-descent (*n* = 10,708). The average participant age was 48.6 years with 53.3% being female. The GWAS results are of rank-based inverse normal transformed protein measures corrected for age, sex, the first ten genome-wide genetic principal components, and test site.

This project focused on combining PWAS that were performed in large cohorts of European descent that had protein levels measured on the same proteomics platform (i.e. the current version of SomaScan). We therefore did not include PWAS conducted on different technologies (e.g. Olink (Folkersen et al. 2017; Sun et al. 2023) or xMAP (Yao et al. 2018)) or previous versions of SomaScan measured in small samples (Emilsson et al. 2018; Suhre et al. 2017; Sun et al. 2018).

#### Outcome GWAS

##### Birthweight

GWAS summary results statistics were obtained from a genetic meta-analysis of birthweight data from the Early Growth Genetics (EGG) consortium and UK Biobank (UKBB) (http://egg-consortium.org/birth-weight-2019.html) (Warrington et al. 2019). We examined four different GWAS meta-analyses performed in individuals of white European descent- own birthweight, offspring birthweight, a GWAS of estimated direct fetal genetic effects on birthweight (i.e. free of maternal influences), and a GWAS of estimated indirect maternal genetic effects on offspring birthweight (i.e. free of fetal influences).

The GWAS of own birthweight involves results from an individual’s own birthweight regressed on their own genotype, whereas the GWAS of offspring birthweight concerns offspring birthweight regressed on maternal genotype. The GWAS of own birthweight was a meta-analysis that combined 35 studies participating in the EGG consortium (*n* = 80,745) and individuals with self-reported birthweight in the UK Biobank (*n* = 217,397). The GWAS of offspring birthweight was a meta-analysis that combined ten studies participating in the EGG consortium (*n* = 12,319) and women who reported the birthweight of their first child in the UK Biobank (*n* = 210,267). For both outcomes, participants that reported multiple births were excluded from the analyses. For both outcomes, for studies that reported gestational age, individuals were excluded if gestational age was less than 37 weeks. For studies that did not report gestational age, individuals were excluded if birthweight was < 2.5 kg or > 4.5 kg (own birthweight), or < 2.2 kg and > 4.6 kg (offspring birthweight). Birthweights were sex-specific z-score transformed and adjusted with available study specific covariates.

The authors in the Warrington et al. (2019) GWAS used structural equation modelling to estimate direct fetal genetic effects on birthweight and indirect maternal genetic effects on birthweight (Warrington et al. 2019). These conditional estimates of the fetal and maternal effects have larger standard errors than the unconditional GWAS analyses of own or offspring birthweight. The corollary is that although these MR analyses should produce asymptotically unbiased results corrected for the effect of fetal (or maternal) genotype, in general they will have lower power than the MR analyses based on the unconditional GWAS.

##### Placental Weight

GWAS summary results statistics for placental weight were sourced from the recent EGG meta-analysis (Beaumont et al. 2023). The placental weight meta-analyses were performed on the fetal, maternal, and paternal genomes with summary statistics from these GWAS representing marginal effect estimates (Beaumont et al. 2023). With contributions from 31 cohorts, the fetal analysis consisted of 65,405 individuals, 61,228 individuals for the maternal and 52,392 for the paternal analyses. All subjects were of European ancestry. Exclusion criteria were pregnancies consisting of multiple births, congenital abnormalities, as well as births earlier than 37 weeks gestation or later than 42 weeks and six days. Placental weight measures were ineligible if they were less than 200 g or greater than 1,500 g or more than 5 standard deviations from the mean. Z-score transformations were performed on placental weight measures within each cohort using unadjusted mean and standard deviation, to account for inter-cohort heterogeneity of collection and measurements.

##### Preeclampsia/Eclampsia

Publicly available summary GWAS statistics on preeclampsia/eclampsia were sourced from Honigberg et al. (2023). The multi-ancestry GWAS meta-analysis was conducted on 17,150 cases (13,893 European, 3,223 Asian, 20 African, and 14 Admixed American) and 451,241 controls (351,259 European, 96,119 Asian, 1,406 African, and 2,457 Admixed American). Cases were classified primarily via International Classification of Diseases (ICD) and phecodes corresponding to preeclampsia and where available, eclampsia. Controls were classified as females that had exclusively normotensive pregnancies or females without codes corresponding to hypertension in pregnancy. The summary statistics for Honigberg et al. (2023) were provided with GRCh38 coordinates and mapped to GRCh37.13.

##### Sporadic Miscarriage

Publicly available GWAS summary statistics of sporadic miscarriage were sourced from Laisk et al. (2020). The GWAS was conducted on 49,996 females of European ancestry who self-reported miscarriages and 174,109 female European controls. Sporadic miscarriage was defined as one or two (self-reported) miscarriages.

#### pQTL Instrument Selection

**Fig. 1:**
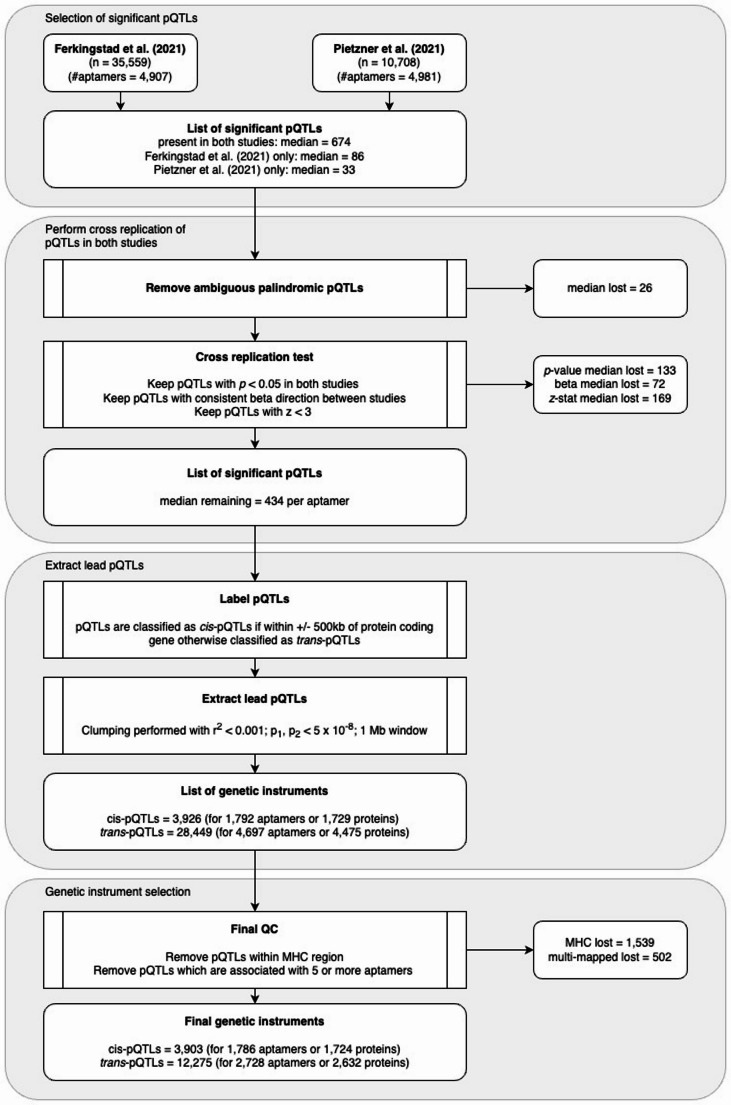
Instrument selection workflow performed to generate *cis*- and *trans*-pQTLs for use as genetic instruments in MR analysis with perinatal outcomes. Median lost refers to the median number of pQTLs lost across all proteins for each instrument selection criteria

Instrument selection was performed by combining the summary statistics of two large proteome GWAS, Ferkingstad et al. (2021) and Pietzner et al. (2021) (Fig. 1). The summary statistics for Pietzner et al. (2021) (Pietzner et al. 2021) were provided with GRCh37 coordinates, whilst all summary statistics from Ferkingstad et al. (2021) were mapped to GRCh37.13. A list of genome-wide significant single nucleotide polymorphisms (SNPs) was created where each SNP was significant (*p* < 5 × 10^−8^) in at least one of the studies. We then removed SNPs that were ambiguous palindromic SNPs (using *TwoSampleMR* R package version 0.5.8 (Hemani et al. 2018) in R version 4.2.1 default settings). A requirement for using the Pietzner et al. (2021) summary results data was that no data were to be used in a meta-analysis. Given this, we instead performed a replication check between the two studies. We performed cross replication of SNPs that were present in both studies, keeping SNPs that replicated (i.e. had *p* < 0.05 and consistent direction of effect between studies) and tested for heterogeneity as determined by a pairwise z-test (keeping SNPs with |Z| < 3). We then split the remaining SNPs into groups of either *cis*- or *trans*-pQTLs. A pQTL was considered a *cis-*pQTL if the pQTL laid within ±500 kb of the protein encoding gene region. All remaining genome-wide significant variants were considered *trans-*pQTLs. Lists of pQTLs for each protein were generated with summary statistics stored from the study that reported the most significant *p*-value. In order to extract independent pQTLs, LD clumping was performed on the replicated pQTL lists for each protein with plink v1.90b3.31 (Purcell et al. 2007) using a threshold of r^2^ < 0.001. The final steps of the instrument selection process involved excluding pQTLs that lay within the human major histocompatibility complex (MHC) region (chr6: from 26 Mb to 34 Mb) and any pQTLs that were associated with five or more aptamers as these were likely to be pleiotropic (Supplementary Table S1).

#### Mendelian Randomisation Analysis

For our primary analysis we only performed MR analyses for proteins that had valid *cis*-pQTLs as instruments as *trans*-pQTLs are likely to represent horizontal pleiotropy. We performed two-sample MR for all proteins that had valid instruments (*n* = 1,724 *cis*-pQTLs and *n* = 1,678 combined *cis*- and *trans*-pQTLs) for the perinatal outcomes of interest using the *TwoSampleMR* R package version 0.5.8 (Hemani et al. 2018) in R version 4.2.1. The primary analysis performed involved *cis*-only MR, where the genetic instruments included were only those classified as *cis*-pQTLs. For pQTLs not present in outcome summary statistics, a proxy (r^2^ > 0.8), from the valid instruments list, was selected where possible. Due to the limited number of *cis*-pQTLs, the MR analyses conducted included the Wald ratio and two-sample inverse variance weighted (IVW) meta-analysis (n_snps_>1) with heterogeneity test (Cochran’s Q-statistic (Bowden et al. 2018). If there were a sufficient number of genetic instruments (n_snps_>5) then additional sensitivity analyses were performed including MR Egger regression (Bowden et al. 2015), weighted median (Bowden et al. 2016), simple mode and weighted mode analyses (Hartwig et al. 2017). Heterogeneity and these additional sensitivity analyses were performed to check for potential violation of the core instrumental variable assumptions due to horizontal pleiotropy. Secondary analysis was performed in a similar fashion which included proteins that had both *cis*- and *trans*-pQTLs available.

#### Genetic Colocalisation

Genetic colocalisation was performed between proteins that had *cis*-only MR results (Wald ratio or IVW) with a *p*-value that passed multiple testing correction (*p*_*Bonferroni*_ < 2.90 × 10^−5^; 0.05/1,724 proteins with *cis*-pQTLs) and the relevant perinatal outcome. Genetic colocalisation was conducted using the *coloc* R package version 5.2.3 (Giambartolomei et al. 2014) as a form of sensitivity analysis to establish if the genetic variants used for MR represented the same causal signal in both the protein exposure GWAS and the perinatal outcome GWAS. Thus, *coloc* essentially tests for violation of the third MR assumption, “exclusion restriction”, by testing for pleiotropic signals due to variants in linkage disequilibrium (LD) with the pQTL being associated with the outcome through pathways other than through serum protein levels. Colocalisation uses a Bayesian framework to estimate posterior probabilities (PP) of the following hypotheses: H_0_ that neither trait has a genetic association in the region; H_1_ that only the protein exposure has a genetic association in the region (which in the context of this study could be due to the outcome cohort being underpowered to detect an effect); H_2_ that only the outcome has a genetic association in the region; H_3_ that both traits are associated with genetic variants in the region but with different causal variants; and H_4_ that both traits are associated and share a single causal variant. Colocalisation assumes a single causal variant in a genomic region. To allow for multiple causal variants, finemapping can first be performed using SuSiE (Sum of Single Effects) with the *SusieR* R package (Wang et al. 2020) to obtain credible sets for use as colocalisation input (Wallace 2021). We performed colocalisation allowing for both a single causal variant (coloc only analysis) and allowing for multiple causal variants in a genomic region (SuSiE and coloc combined analysis) for proteins that had MR findings passing multiple testing correction for an outcome. Finemapping with SuSiE was performed with 0.95 coverage threshold, ensuring there is a 95% probability a credible set contains a causal variant. For both coloc only analyses and SuSiE and coloc combined analyses, the default prior probabilities were used, with *p*_*1*_ = *p*_*2*_ = 1 × 10^−4^ where *p*_*1*_ is the prior probability that each variant is causal for the protein exposure and *p*_*2*_ is the prior probability that each variant is causal for the perinatal outcome and *p*_*12*_ = 1 × 10^−5^ where *p*_*12*_ is the probability that each variant is causal for both traits (Giambartolomei et al. 2014). A PP > 0.8 for H_4_ was considered strong evidence of a shared causal signal between the protein exposure and perinatal outcome. Proteins and perinatal outcomes that had significant *cis*-only MR results (*p* < 2.90 × 10^−5^) and evidence of colocalisation (H_4_ PP > 0.8) were considered strong potential genetic associations.

## Results

### Summary of Genetic Instruments

Following instrument selection, there were 1,724 proteins with genome-wide significant *cis*-pQTLs (Supplementary Table S2). Of the proteins that had genome-wide significant *cis*-pQTLs, 1,324 proteins additionally had genome-wide significant *trans*-pQTLs. All pQTLs excluded due to associations with multiple proteins were *trans*-pQTLs (100%) with a few of these also being *cis*-pQTLs (3.19%).

### Mendelian Randomisation and Colocalisation

#### Summary of Mendelian Randomisation Results

We investigated the potential causal relationship between circulating proteins (n_proteins_=1,724) using MR and a range of adverse perinatal outcomes, conducting both a *cis*-only MR analysis (Supplementary Tables S3–12) and a *cis*- and *trans*-combined analysis (Supplementary Tables S13–22). For the most part, the combined *cis*- and *trans*-analysis produced similar directions of effect for the causal estimate albeit with a smaller magnitude and less significant *p*-value. In total, ten different perinatal outcomes were included in the MR analyses, with there being six outcomes in which evidence for causal relationships were detected after Bonferonni correction for multiple testing (Supplementary Tables S3–12). These results are highlighted below.

#### Offspring Birthweight

**Fig. 2:**
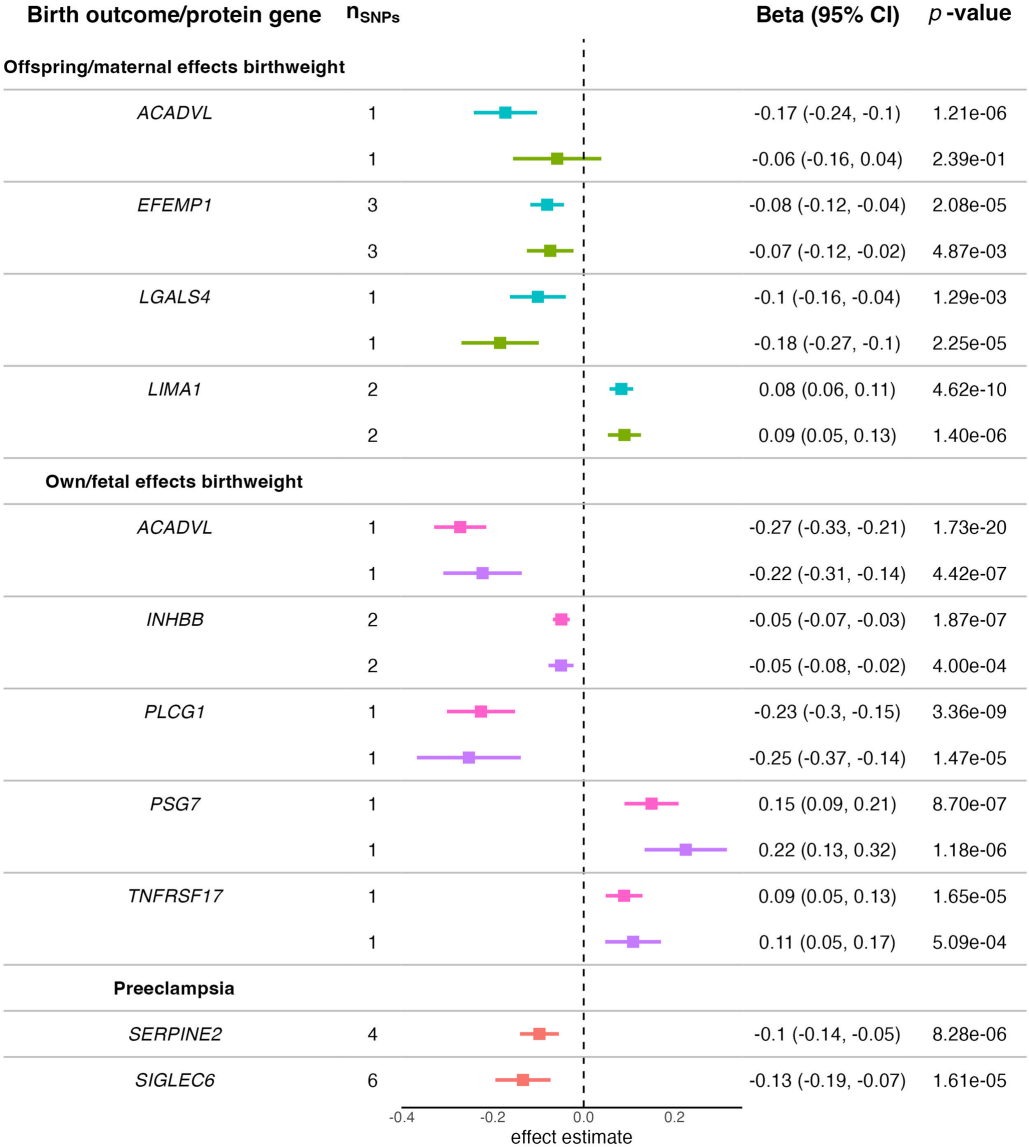
Two-sample MR results that passed multiple testing correction (*p* < 2.90 × 10^−5^) for the causal effect of protein levels on offspring birthweight (blue and green), own birthweight (pink and purple), and preeclampsia (red). The green and purple causal estimates are those obtained using conditional analyses for the SNP-birthweight association. The overview includes the MR causal effect estimate, given by either the Wald ratio (n_SNPs_=1) or IVW MR (n_SNPs_>1), with the 95% confidence interval (CI). Birthweight was analysed as a z-score, preeclampsia as a log-odds ratio, and proteins were analysed as inverse normal transformations of protein levels (Color figure online)

We investigated potential causal relationships between maternal circulating proteins (n_proteins_=1,724) and offspring birthweight. Ten proteins showed potential causal effects on offspring birthweight after correction for multiple testing (Supplementary Tables S3–S4; Fig. 2). Specifically, our analyses suggested that elevated levels of circulating proteins encoded by *LIMA1*, *NPPB*, and *PRSS8*, and decreased levels of circulating proteins encoded by *ACADVL*, *CSK*, *CTRB2*, *EFEMP1*, *SEC22A*, and *ULK3* were causal for increased offspring birthweight. Causal effect estimates for proteins that had more than one valid *cis*-pQTL available as an instrument did not show significant evidence of heterogeneity (Q *p*-value; *CTRB2* = 0.2025, *EFEMP1* = 0.9639, *LIMA1* = 0.6258, and *PRSS8* = 0.9106). Findings were similar when using estimated maternal genetic effects on offspring birthweight as the outcome with the exception of *EFEMP1*. Of the nine aforementioned maternal circulating proteins potentially causal for offspring birthweight, four (encoded by *ACADVL*, *EFEMP1*, *LGALS4*, and *LIMA1*) showed strong evidence of colocalisation (PP H_4_ > 0.8; *ACADVL*, *LGALS4*, and *LIMA1*), or inconclusive evidence suggesting an underpowered outcome (PP H_1_ > 0.8; *EFEMP1*) (Supplementary Table S23).

#### Own Birthweight

We investigated potential causal relationships between fetal circulating proteins (n_proteins_=1,724) and birthweight. Eight proteins showed potential causal effects on birthweight after correcting for multiple testing (Supplementary Tables S5–S6; Fig. 2). Our analyses suggested that elevated levels of circulating PSG7 and TNFRSF17 and decreased levels of circulating proteins encoded by *ACADVL*, *INHBB*, *LILRB3*, *MAPRE1*, *PLCG1*, and *TNFRSF6B* were causal for increased (own) birthweight. Causal effect estimates for the two circulating proteins that had more than one valid *cis*-pQTL available as an instrument did not show evidence of heterogeneity (Q *p*-value; *INHBB* = 0.9785 and *LILRB3* = 0.1207). Findings were similar when using estimated fetal genetic effects on own birthweight as the outcome for all eight proteins. Of the eight aforementioned fetal circulating proteins potentially causal for own birthweight, five (encoded by *ACADVL*, *INHBB*, *PLCG1*, *PSG7*, and *TNFRSF17*) showed strong evidence of colocalisation (PP H_4_ > 0.8) with birthweight (Supplementary Table S24).

#### Placental Weight

Increased circulating levels of LIMA1 in the fetus were potentially causal for increased placental weight (0.11 SD increase in placental weight per SD increase in serum levels of LIMA1). There were two independent *cis*-pQTLs available as instruments for LIMA1 and both provided homogenous estimates for the causal effect on placental weight (Q p-value; LIMA1 = 0.6953). However, colocalisation analyses were inconclusive making it unclear whether genetic associations for LIMA1 levels and placental weight represented the same underlying signal (Supplementary Table S25).

#### Preeclampsia

Two proteins had a potential causal effect on risk of preeclampsia after correction for multiple testing (Supplementary Tables S10; Fig. 2). Analyses suggested that decreased levels of circulating proteins encoded by *SERPINE2* and *SIGLEC6* were causal for increased risk of preeclampsia. Causal effect estimates for both proteins did not show evidence of heterogeneity when considering different *cis*-pQTLs (Q *p*-value; *SERPINE2* = 0.5880 and *SIGLEC6* = 0.9882). Furthermore, *SIGLEC6* had enough *cis*-pQTLs as valid instruments (n_SNPs_=6) to perform further sensitivity analyses. Causal estimates produced by the different methods had consistent direction of effect compared to the IVW estimate and all save MR-Egger were nominally significant (i.e. *p* < 0.05). *SERPINE2* showed strong evidence (PP H_4_ > 0.8) of sharing the same causal variant with the birthweight outcome, whilst *SIGLEC6* showed inconclusive evidence suggestive of an underpowered outcome (PP H_1_ > 0.8) (Supplementary Table S26).

## Discussion

We used MR to investigate potential causal relationships between circulating proteins (n_proteins_=1,724) and a range of adverse perinatal outcomes. We identified five circulating proteins (encoded by *ACADVL*, *INHBB*, *PLCG1*, *PSG7*, and *TNFRSF17*) potentially causal for own birthweight, and four circulating proteins (encoded by *ACADVL*, *EFEMP1*, *LGALS4*, and *LIMA1*) potentially causal for offspring birthweight. We also identified two circulating proteins (encoded by *SERPINE2* and *SIGLEC6*) as potentially causal for preeclampsia. We did not find any significant effects of proxied circulating proteins on sporadic miscarriage, perhaps due to the smaller size of its GWAS meta-analysis. We discuss some of the more interesting results in the main text and summarise our other significant findings in Supplementary Table S27, S28.

Our results suggest that decreased levels of INHBB, the inhibin subunit B chain of the heterodimeric glycoprotein inhibin B, (Namwanje and Brown 2016), may be causal for increased birthweight. Inhibin B is an endocrine protein and member of the transforming growth factor beta (TGFβ) superfamily, which is produced in the gonads (both ovaries and testes) and acts as an antagonist to activin, regulating the synthesis and secretion of follicle-stimulating hormone (FSH) (Henry and Norman 2003). Circulating levels of inhibins are known to fluctuate during the menstrual cycle as well as throughout pregnancy. Whilst a previous small scale GWAS of preeclampsia implicated SNPs in close proximity to the *INHBB* genomic region in the etiology of the condition (Johnson et al. 2012), subsequent GWAS meta-analyses failed to replicate these findings (Honigberg et al. 2023). Indeed, our own MR findings suggest no causal relationship between circulating INHBB and preeclampsia (Supplementary Table S10, *p* = 0.1470).

The protein PSG7, pregnancy specific beta-1-glycoprotein 7, is a member of the pregnancy specific glycoprotein (PSG) family which is predominantly expressed during pregnancy by placental trophoblasts and secreted into the maternal circulation (Moore et al. 2022). PSGs are also expressed at low levels in a range of non-placental tissues unrelated to pregnancy. Proposed roles for PSGs during pregnancy include regulating maternal immune and vascular functions or acting locally in the placental bed with autocrine and paracrine functions. Generally speaking, the expression of PSGs appears to be highly conserved amongst species with hemochorial placentation. Elevated levels of circulating maternal PSGs during pregnancy are thought to be primarily driven by the fetus (Moore et al. 2022). Our findings suggest that decreased levels of PSG7 are causal for increased (own) birthweight. Previous clinical cohort studies have implicated increased levels of circulating PSG7 and PSG9 with preeclampsia (Kandel et al. 2022). Our MR findings reported a similar direction of effect with PSG7 having a nominally significant *p*-value for preeclampsia (Supplementary Table S10, *p* = 0.0345).

The *SERPINE2* gene encodes protease nexin 1 (PN-1) a member of the serin protease inhibitor (*SERPIN*) gene family. Our findings suggest that decreased levels of circulating *SERPINE2* cause increased risk of preeclampsia. *SERPINE2* is widely expressed across different tissues including the placenta, and is implicated in tissue remodelling during implantation (Chern et al. 2011). Within the context of preeclampsia, studies of *SERPINE2* expression have been mixed e.g. Chelbi et al. (2007) (Chelbi et al. 2007) reporting that hypoxia induced the expression of *SERPINE2*, whereas Sheridan et al. (2019) (Sheridan et al. 2019) reported upregulation of *SERPINE2* in the absence hypoxia. Our results for decreased circulating PN-1 levels associated with an increased risk of preeclampsia mirrors previously reported MR results (Ardissino et al. 2024). Ardissino et al. (2024) (Ardissino et al. 2024) additionally reported that *SERPINE2* pQTL results were corroborated by whole blood eQTLs.

The SIGLEC6 gene encodes a protein that binds to sialic acid and leptin (Patel et al. 1999). It is a member of the SIGLEC (sialic acid binding immunoglobulin-like lectin) family of proteins and expressed in trophoblasts of the human placenta. Siglec-6 is consistently overexpressed in placentas from pregnancies complicated by preeclampsia compared to controls (Enquobahrie et al. 2008; Sitras et al. 2009; Tsai et al. 2011; Winn et al. 2009). This raises the interesting question of whether Siglec-6 may be part of the causal pathway in pre-eclampsia. Interestingly, our pQTL results are consistent with a causal relationship, however, our results imply the opposite direction of effect (i.e. increased serum levels of SIGLEC6 are associated with decreased risk of preeclampsia). It is unclear why this might be the case but may reflect that we are proxying serum levels of the protein as opposed to levels in the placenta.

There are a number of limitations to our study, primarily concerned with the measurement and reliability of the proxied protein levels. Firstly, protein levels are known to vary across cell types and tissues (Uhlén et al. 2015). As our study focused solely on protein levels measured in circulating blood, we are unlikely to detect protein level changes specific to tissues other than blood.

Second, current protein assay methods including the SomaScan technology utilized in the pQTL GWAS the present manuscript drew on, have the potential to be subject to epitope effects. Epitope effects arise when the genetic variant (or one in linkage disequilibrium with it) alters the assay binding site (and consequently measured levels of the protein) but otherwise has no genuine functional effect on the protein. In other words, epitope effects represent spurious pQTLs due to a technical artifact. In general, *cis*-pQTLs are more prone to epitope effects than *trans*-pQTLs. In the context of the present study, the presence of epitope effects may lead to the misattribution of causality to serum protein levels if there also happens to be a genetic signal at the same variants in the perinatal trait (outcome) GWAS. Nevertheless, colocalization analyses should provide some protection against this potential artifact as the pattern of genetic association in the region should differ between the protein and perinatal trait GWAS.

Third, our investigation was limited to *cis*-pQTLs that showed robust association with circulating protein levels. In general, *cis*-pQTLs are more likely to explain larger proportions of the variance in serum protein level than trans-pQTLs, and to generate causal estimates that are uncontaminated by genetic pleiotropy. We chose to exclude *trans*-pQTLs from most analyses as they are more likely to be pleiotropic, therefore violating one of the core assumptions of MR. However, in focusing only on *cis*-pQTLs, we had limited numbers of genetic variants to proxy each protein, in most cases removing the ability to perform further MR sensitivity analyses.

Fourth, the proteins examined in this study were proxied using the results from a population-based GWAS which included males and (non-pregnant) females. Given our focus on adverse perinatal outcomes, it is of note that the protein levels of these studies were not measurements taken from a mother or fetus during pregnancy. We have therefore made the assumption that pQTLs that proxy serum protein levels in the general population will also proxy serum protein levels in the fetus and mother during pregnancy. Future proteome wide studies will need to be conducted in populations of pregnant women to confirm this assumption.

Fifth, we would also like to acknowledge some degree of sample overlap between the Pietzner et al. (2021) protein GWAS (*n* = 10,708) and the Warrington et al. (2019) birthweight GWAS meta-analysis (*n* = 210,267–298,142). However, bias generated by sample overlap is only a concern in the case of weak instruments. It is unlikely that any of the pQTLs used in our study were weak instruments given the strict criteria around instrument selection (i.e. *cis*-pQTLs reaching *p* < 5 × 10⁻⁸), and therefore any resulting bias is likely to have been small.

Finally, whilst some of the perinatal GWAS we drew on involved hundreds of thousands of participants (e.g. birthweight), others were relatively small by current standards (e.g. preeclampsia). The corollary is that we may not have been well powered to detect causal relationships between serum protein levels and these latter traits.

Our results implicate several proteins that may be involved in the aetiology of birthweight and that will need to be replicated in independent datasets. We expect that as the volume of summary results GWAS data from national biobanks and international GWAS consortia increases over the coming years, that pQTL scans like ours will yield rich rewards in terms of understanding the aetiology of adverse perinatal outcomes.

## Supplementary Information

Below is the link to the electronic supplementary material.

Supplementary Material 1

## Data Availability

No datasets were generated or analysed during the current study.
